# Profiling hearing aid users through big data explainable artificial intelligence techniques

**DOI:** 10.3389/fneur.2022.933940

**Published:** 2022-08-26

**Authors:** Eleftheria Iliadou, Qiqi Su, Dimitrios Kikidis, Thanos Bibas, Christos Kloukinas

**Affiliations:** ^1^1st Department of Otorhinolaryngology-Head and Neck Surgery, National and Kapodistrian University of Athens Medical School, Athens, Greece; ^2^Department of Computer Science, University of London, London, United Kingdom

**Keywords:** explainable AI (XAI), Deep Learning, big data, hearing loss, Hearing Aids, prognosis prediction, Long Short-Term Memory (LSTM), attention mechanism

## Abstract

Debilitating hearing loss (HL) affects ~6% of the human population. Only 20% of the people in need of a hearing assistive device will eventually seek and acquire one. The number of people that are satisfied with their Hearing Aids (HAids) and continue using them in the long term is even lower. Understanding the personal, behavioral, environmental, or other factors that correlate with the optimal HAid fitting and with users' experience of HAids is a significant step in improving patient satisfaction and quality of life, while reducing societal and financial burden. In SMART BEAR we are addressing this need by making use of the capacity of modern HAids to provide dynamic logging of their operation and by combining this information with a big amount of information about the medical, environmental, and social context of each HAid user. We are studying hearing rehabilitation through a 12-month continuous monitoring of HL patients, collecting data, such as participants' demographics, audiometric and medical data, their cognitive and mental status, their habits, and preferences, through a set of medical devices and wearables, as well as through face-to-face and remote clinical assessments and fitting/fine-tuning sessions. Descriptive, AI-based analysis and assessment of the relationships between heterogeneous data and HL-related parameters will help clinical researchers to better understand the overall health profiles of HL patients, and to identify patterns or relations that may be proven essential for future clinical trials. In addition, the future state and behavioral (e.g., HAids Satisfiability and HAids usage) of the patients will be predicted with time-dependent machine learning models to assist the clinical researchers to decide on the nature of the interventions. Explainable Artificial Intelligence (XAI) techniques will be leveraged to better understand the factors that play a significant role in the success of a hearing rehabilitation program, constructing patient profiles. This paper is a conceptual one aiming to describe the upcoming data collection process and proposed framework for providing a comprehensive profile for patients with HL in the context of EU-funded SMART BEAR project. Such patient profiles can be invaluable in HL treatment as they can help to identify the characteristics making patients more prone to drop out and stop using their HAids, using their HAids sufficiently long during the day, and being more satisfied by their HAids experience. They can also help decrease the number of needed remote sessions with their Audiologist for counseling, and/or HAids fine tuning, or the number of manual changes of HAids program (as indication of poor sound quality and bad adaptation of HAids configuration to patients' real needs and daily challenges), leading to reduced healthcare cost.

## Introduction

Hearing Loss (HL) is a public health problem that affects one out of three people over the age of 65, while debilitating HL is estimated to affect 6% of the population (466 million people) according to World Health Organization (WHO) statistics^1^. As per the same statistics, its annual management cost is estimated at more than 555 billion Euros (1) for the European countries and at 750 billion Dollars globally. HL should not be considered as an isolated health problem. Apart from the associated financial cost, HL severely affects communication and is associated with various comorbidities. Multiple studies have suggested that hearing impairment is associated with psychological and physical illness, such as cognitive disorders and dementia. An increase in the hearing threshold of 25 decibels (dB) corresponds to a loss of 7 cognitive years (2), and is associated with increased anxiety and depression (3), and even higher mortality rate (4). On the other hand, adults with hearing impairment tend to isolate themselves by limiting their participation in social events (5), thereby reducing their quality of life significantly (6).

Although the only available and validated management solution that currently exists for HL is the fitting and use of hearing assistive devices, only one in five people in need of a Hearing Aid (HAid) will eventually seek, acquire, and continue to use one efficiently (7, 8). A “HAid experience” refers to the process of living with a HAid and involves all the real-life challenges, coping strategies, and facilitations that the uses of HAid may evoke. Improvements in the HAid experience can lead to minimization of drop-out risk and enhancement of the overall quality of life (9).

The key factors in improving the HAid experience include, but are not limited to, proper fitting, affordability and accessibility of the follow-up services, and their combination with thorough and evidence-based personalized counseling and training on how to use the selected HAid (10). Since everyday patient needs and HL degree are not static and might change over time, there are still many factors that audiologists find challenging to address, including selecting optimal HAid configurations or best counseling approach according to individual patient profile and lifestyle (7, 11–13). Dynamic monitoring and collecting information about a patient's hearing and cognitive capacity, as well as their ability to control settings in real time in order to cope in different sound environments, could be very helpful toward this direction (14, 15). The development and validation of prediction models using the collected information and making accurate prognoses of how each patient's HAid experience will unfold are of major priority.

The use of Artificial Intelligence (AI) models in prognosis studies has gained traction increasingly in recent years due to its ability to handle large amounts of messy data (16), to learn from different types of data (17), and to facilitate clinical management of patients (18). Researchers have incorporated AI models in prognosis in clinical cancer research, such as breast cancer with Support Vector Machine (SVM) (19), colorectal cancer with Long Short-Term Memory (LSTM) (20), and glioblastoma with Prognosis Enhanced Neural Network (PENN) (21). As well as the prognosis for adult congenital heart disease with Convolutional Neural Network (CNN)-LSTM (22), rate of kidney disease with an ensemble of Logistic Regression, Decision Tree, Random Forest (RF), and K-Nearest Neighbor (KNN) (23), and COVID-19 with a segmentation network (24).

The effectiveness of AI models in HL prognosis has also been investigated by many researchers. Sensorineural Hearing Loss (SNHL) is the most common form of permanent HL resulting from the damage to the auditory nerve and/or the hair cells in the inner ear. Abdollahi et al. (25) constructed eight Machine Learning (ML) models to predict SNHL after chemoradiotherapy, including Decision Stump, Hoeffding, C4.5, Bayesian Network, Naïve, Adaptive Boosting (AdaBoost), Bootstrap Aggregating, Classification *via* Regression, and Logistic Regression (LR). The average predictive power of all models was found to be more than 70% in terms of accuracy, precision, and Area Under Curve (AUC). Idiopathic Sensorineural Hearing Loss (ISSHL) is characterized by an acute dysfunction of the inner ear. Zhao et al. (26) developed several ML models for ISSHL prediction, including SVM, Multilayer Perceptron (MLP), RF, and AdaBoost. A similarly high level of accuracy is also reported and varies between 78.6 and 80.1%. Bing et al. (27) evaluated several Deep Learning (DL) and ML models to predict the dichotomised hearing outcome of ISSHL in order to identify the best predictive model for clinical application. Six input feature collections derived from 149 potential predictors have been used with Deep Belief Network, LR, SVM, and MLP. Best predictive performance was achieved by Deep Belief Network when evaluated with accuracy, precision, recall, F-score, Receiver Operating Characteristic Curve (ROC), and AUC, achieving 77.58% of accuracy and 0.84 of AUC. Ototoxic-induced HL, more specifically, the ototoxic effects in participants who were exposed to cigarette smoke and/or pesticides were evaluated by Artificial Neural Network, KNN, and SVM (28). While all models showed a good performance during training, KNN achieved the highest training accuracy with about 90% in two of the five datasets.

Attention-based DL models have also gained popularity in the medical domain recently. Bahdanau et al. (29) proposed the first attention mechanism, also known as the Soft Attention, for a Neural Machine Translation task using LSTM. The advantage of using attention mechanisms with LSTM is that it prevents the LSTM from forgetting certain input features when analyzing long-term dependencies and from putting too much weight on certain input features. Despite the lack of research using attention-based LSTM for HL patients specifically, a similar approach has been adapted for other comorbidities. Park et al. (30) used a Frequency-aware Attention-based LSTM (FA-Attn-LSTM) to investigate medical features that can be considered as critical for predicting the risk of cardiovascular disease. Wall et al. (31) proposed a framework for audio classification, specifically for chronic and non-chronic lung disease and COVID-19 diagnosis, with attention-based bidirectional LSTM (A-BiLSTM).

AI, particularly DL models, in general are appreciated for their ability to achieve high prediction accuracy. However, for sensitive domains, such as health care, accuracy is not the only determining factor (32). The inherent limitation of many AI systems is their black box nature, which means that humans are unable to easily understand the inner workings of these systems or how they arrive at their conclusions. Thus, automated decision-making systems that employ AI models are not widely accepted (32) due to a lack of trust from the end users. The integration of AI models into medical domains also faces criticisms where the models may fail to adhere to high standards of accountability, reliability, and transparency for medical decisions (33). It also complicates the issue of accountability in the event of a wrong decision (34).

Explainable AI (XAI) aims to overcome these limitations by explaining the learned decisions of AI models, thus giving end-users the ability to trust the models (35) and understanding why the models made certain decisions (32). Different XAI methods have been proposed over the years, particularly in the fields of computer vision and natural language processing. Yet very few studies have explored the potential applications of XAI methods to the medical field (34), especially in prognosis studies. A number of researchers have adapted Local Interpretable Model-agnostic Explanation (LIME) (36) to explain a CNN-based diagnostic model, including chronic wound classification (37), gastral image classification (38), and Alzheimer's diagnosis (39). Gu et al. (40) proposed an auxiliary decision system for breast cancer diagnosis and prediction with Extreme Gradient Boosting (XGBoost) and SHapley Additive exPlanations (SHAP) (41). Chakraborty et al. (17) developed a similar framework that was inspired by Gu et al. (40) using XGBoost and SHAP for prognosis in breast cancer patients. In the HL domain, Lenatti et al. (42) applied SHAP to explain the classification results of RF in predicting whether or not a patient has HL. In particular, SHAP is used to investigate the local predictions for each of the two output classes in four scenarios: true positive, true negative, false positive, and false negative. They have found that Age is the most important feature that impacts the classifier. In particular, values of age equal to 74 contribute positively to the model correctly predicting participants with HL (true positive), whereas values of age equal to 25 contribute negatively to the model correctly predicting participants without HL (true negative).

To the best of our knowledge, this is the first conceptual paper on a framework that leverages AI and XAI for prognosis for HL benefit and usage. ML techniques have been implemented previously in studies focusing on the prognosis of SNHL, ISSHL, and HL induced by ototoxic drugs and other substances (25–28), and modeling has also been attempted with synthetic data in more progressive types of HL, such as age-related or noise-induced HL (43). Nevertheless, we are unaware of any such attempts with real multi-source big data to date.

In the EU-funded SMART BEAR project^2^, we are developing and validating a prognosis framework to address this scientific gap for HL patients. AI and XAI techniques will help identify and explain particular trends and factors in the large amount of heterogeneous data collected that correlate with the success or failure of hearing rehabilitation. In particular, the proposed framework composes the predictive power of LSTM with Attention Mechanism with the explanatory abilities of SHAP, and it will be used to answer several questions to provide a comprehensive profiling of HL patients.

The purpose of this article is to describe the planned data collection process, as well as the upcoming analyses to identify and explain particular trends and factors that correlate with the success or failure of hearing rehabilitation: drop-out of HAids usage, more hours of HAids usage and higher benefit from it, and less frequent need for manual adjustments or fine tuning of the HAids. As this is a conceptual paper, data collection is expected to begin in autumn 2022, followed by the experiments of the proposed methods.

## Materials and methods

### Participants

Five thousand elderly participants from six different EU countries will be included in the study. In particular, these six countries are divided into five study groups and 1,000 participants are recruited from each, namely France, Greece, Italy, Romania, and Portugal-Spain. A smaller-scale pilot study with 100 participants is already underway in the island of Madeira. The large-scale project is scheduled to begin in autumn 2022 and run for 24 months. Subjects will be included in the study based on the following eligibility criteria:

1. Age and birth gender: males and females, 67–80 years old.
2. Medical history: at least 2 of the following conditions: cardiovascular diseases (CVDs: hypertension, coronary disease, heart failure), hearing loss, balance disorders, mild depression, mild cognitive impairment, frailty.
3. Cognitive function according to MoCA score: participants with 26–30/30 (no cognitive impairment), and 18–26/30 (mild cognitive impairment) will be included (44). Score lower than 18/30 corresponds to mild dementia which is not addressed in SMART BEAR so those participants scoring <18/30 will be excluded.
4. Excellent to Moderate level of mobility, which corresponds to be able to perform simple tasks such as walking and jumping independently, with or without the help of a mechanical equipment, for example, a cane.
5. Ability to read.
6. Ability to use the basic functions of a smartphone (answer, call, check a notification, open an application).

**Figure 1:**
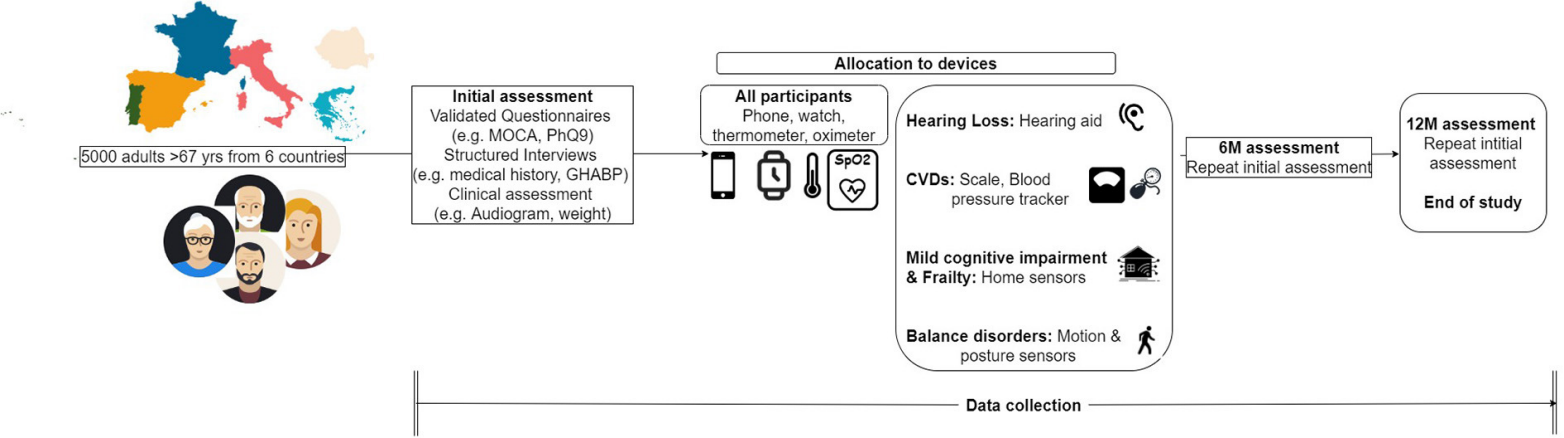
Participants' flow of action.

Participants who meet the aforementioned criteria but present a severe or life-threatening condition, such as severe depression or high risk of heart failure, will be excluded from the study. All participants willing to provide their informed consent and voluntarily participate in the study will undergo an initial clinical assessment as shown in Figure 1. According to the results of this screening assessment, a specific set of devices and clinical procedures will be allocated to each participant. These devices are being obtained through joint procurement for all six countries and will be the same in terms of type, model, and configuration for all participants.

### Participants with hearing loss

We intend to recruit one thousand people with HL to a degree that requires amplification. Participants with a moderate to severe unilateral or bilateral HL, as indicated by their pure tone audiogram, are considered eligible for HAid fitting if their HL negatively impacts their communication ability, cannot be treated surgically, or can be treated but the surgery is contra-indicated for the particular participant. Participants will only be excluded from Fitting if they do not wish to be fitted with a HAid, or if they have profound HL (Pure tone average 0.5–4 kHz > 80 dB), and have not received any benefit from recent previous HAid fitting and use.

### Audiological assessment

**Figure 2:**
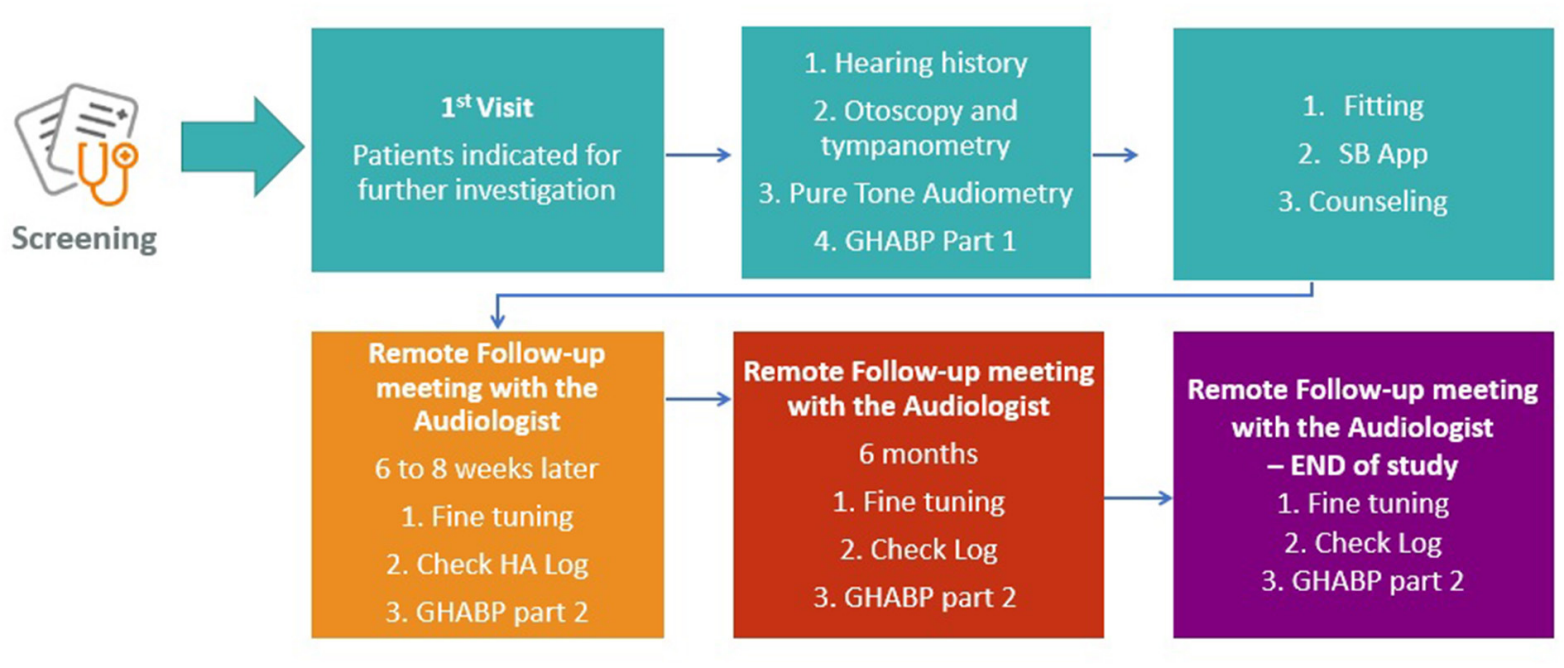
Audiological assessment flow of action.

The same audiometric assessment (Figure 2) will be conducted on all participants with suspected or diagnosed HL by experienced personnel who have undergone additional internal training on every procedure of the clinical protocol by the clinical coordination team of the SMART BEAR. Joint procurement will ensure that the equipment (including HAids) and relevant software will be the same for all countries. Following the audiometric assessment, all participants will be fitted with HAids according to the same fitting protocol. The exact fitting protocol will be defined once the specific model and manufacturer of the HAids is selected during the international procurement procedure as discussed above. HAids configuration will then be fine-tuned in accordance with the participant's experience level, listening preferences, and language preferences. There will be a predefined HAids program for all participants, other programs may be added based on the judgment of the audiologists and the needs of the participants. Pure tone audiometry will follow the British Society of Audiology^3^ guidelines.

In accordance with the SMART BEAR fitting protocol, participants will be monitored for 12 months after they have been fitted with either one or two HAids (same manufacturer, same model). As shown in Figure 2, participants will also have continuous access to remote and face-to-face fine-tuning services provided by the SMART BEAR audiologists. Through the SMART BEAR clinician's dashboard, the audiologists will have access to participants' data and HAids log throughout this period.

### Source of data

**Figure 3:**
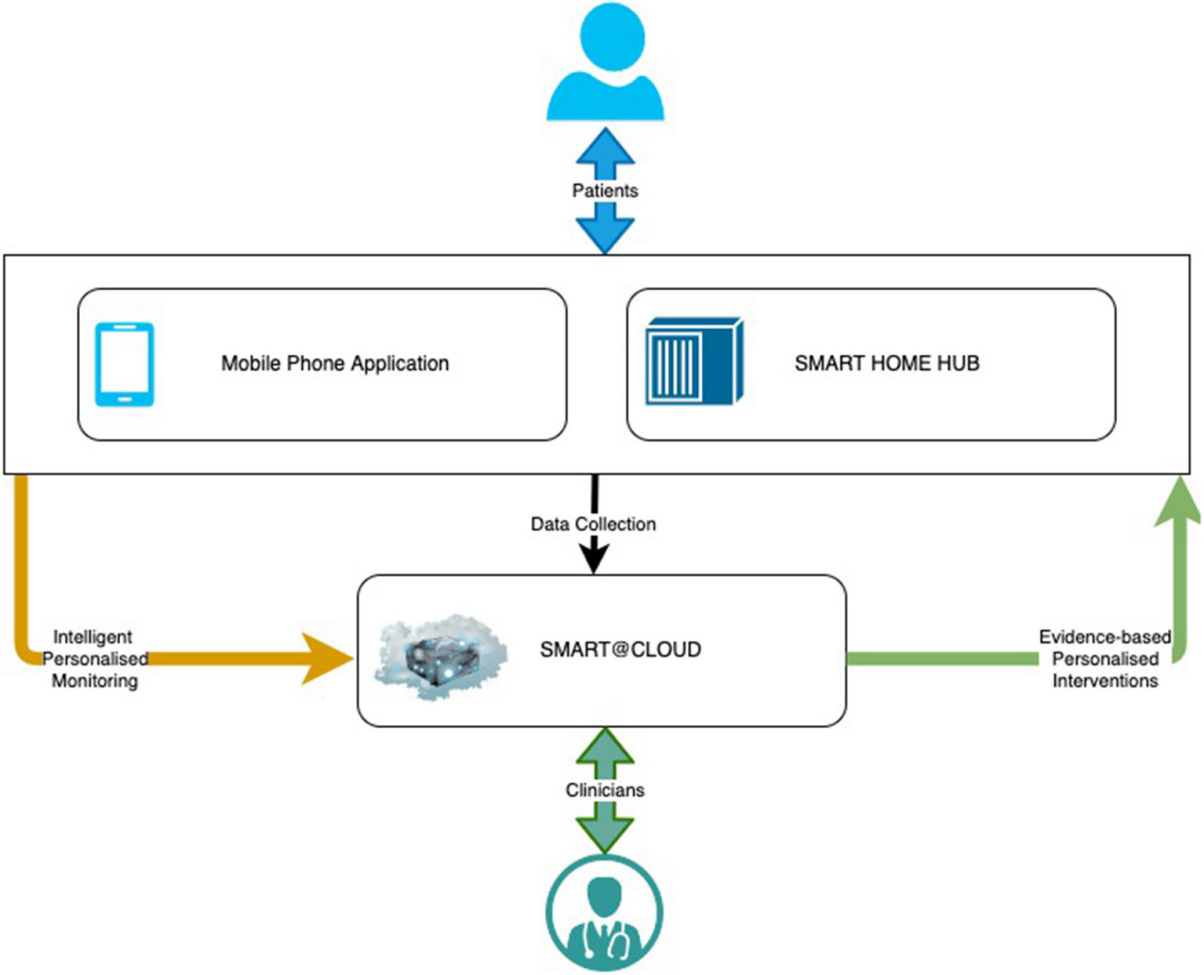
The SMART BEAR architecture.

SMART BEAR is a large-scale multi-centric clinical study that aims to integrate state-of-the-art technology into everyday life of senior citizens with specific comorbidities, composing off-the-shelf and user-friendly devices onto an innovative platform. There are three subsystems in the SMART BEAR architecture as shown in Figure 3, namely the mobile phone application, the SMART BEAR HomeHub, and the SMART BEAR Cloud (SB@Cloud). Data are collected (i) during participants' clinical assessments *via* the clinician dashboard (e.g., anamnestic history, physiological and audiometric measurements), (ii) from all linked portable devices *via* the mobile phone application (e.g., HAid program, heart rate, and steps measurement), and (iii) through the mobile phone application itself (e.g., through questionnaires about their mood, diet, medication adherence and sleep quality). The HomeHub accumulates data from different home-based device sensors, such as weight scales and movement sensors. Finally, SB@Cloud securely stores and analyses the collected data through model and data-driven big data analytics during a 12-month period for each participant.

A total of 24 variable and covariates are collected through SMART BEAR HAids, including timestamp of the measurement, environmental noise, and manual program adjustments. Supplementary Table 1 provides a detailed description of each variable and covariate. Several other covariates are also being considered and are shown in Supplementary Table 2. The additional 241 covariates are collected in order to monitor the participants' other comorbidities based on their demographics, biological, environmental, and behavioral characteristics. There is a need to consider the impact of these additional covariates on the outcomes since they have been previously shown to affect to HL and HAid experiences, such as age, occupation, education, family history, mood disorders, cognitive function, diet, glucose levels and medication (3, 45–47). They are also currently being investigated for their correlation to hearing, as in the case of cardiovascular diseases, poorer mobility, frailty, and balance disorders (46, 48, 49). Furthermore, the medical and audiological assessment will also be supplemented by additional sensor data as listed in Supplementary Table 3, such as blood pressure measured by the blood pressure tracker and physical activity measured by the smart watch. These variables are collected as a part of SMART BEAR's commitment to collect a wide range of data which will be explored as a part of data-driven analysis.

### Sample size

SMART BEAR is aiming at collecting and analyzing big data—integrating information from many thousands of participants and different data sources. In Big Data, common sample size calculations cannot apply (50). Big data studies need to consider the marginal costs vs. the marginal value of possible sample sizes and include as many participants as possible (51). In SMART BEAR, the maximum number of participants that can be recruited based on available resources and time is 5,000. In accordance with the requirements of the study, this number is considered sufficient for ensuring the impact analysis obtained at the end of the project to be significant. In the case of HL, 200 participants with HL will be recruited from each of the five study groups, creating a sample of 1,000 participants with HL. These participants will then be fitted with either one or two HAids depending on whether one or both ears require amplification. Therefore, the total number of HAids to be used in the planned data collection is estimated between 1,000 and 2,000. The SMART BEAR platform is designed to facilitate the collection of data from a maximum number of 2,000 HAids, in case all participants suffer from bilateral HL. Data collected from up to 2,000 HAids are also considered to be sufficient based on previous experience (50).

### Analysis methods

The questions that will be addressed with the proposed framework are based on future events. The prediction model will be used, for example, to predict future HAid usage or future drop-out rate. As a result, the model is fundamentally constructed with participants' historical medical history, HAid usage and habit, as well as the outcomes of medical and audiological assessments. As such, the collected SMART BEAR data are sequential in nature and can be viewed as time series data.

The proposed framework uses an attention-based LSTM (attn-LSTM) as the prediction model and then applies SHAP to interpret the model predictions. More specifically, SHAP is employed to identify those characteristics that influence the model predictions. To enable continuous learning and provision of personalized solutions, the pipeline for the proposed framework is to pre-process the data, hyper-tune the model, train/test the model with the optimal set of hyper-parameters selected from hyper-tuning, and then apply the XAI method. The performance of the prediction models is evaluated using different set of evaluation metrics for classification and regression problems.

#### Pre-processing the data

The temporal element of the collected data is determined by the Time variable, which records the date and time of the collected variables every 60 s when the SMART BEAR HAids are active in use. In SMART BEAR, clinicians also have the option of choosing how the data are aggregated for different analysis. Due to this, the data frequency is transformed first in order to allow hourly, daily, weekly, monthly, or yearly predictions, depending on the choice of clinician.

Transforming the distribution of the features allows the ML and DL algorithms to converge faster and minimize the weight of any variable with extreme values. *Standardization* and *normalization* are two pre-processing techniques that are particularly important for training an LSTM algorithm, since standardization on the data centers the noise from trend reverse signals and prevents activation functions to saturate (52), whereas normalization prevents the weights of the model being skewed (53).

Ordinal variables will be transformed with ordinal encoding and nominal variables will be transformed with one-hot encoding in order to convert these variables into either binary or multiple values with a numerical form. If the expected outcome variable is categorical then these will be treated label encoding.

Another important pre-processing step is to handle missing data. Several studies regarding data completeness in medical data were reviewed by Chan et al. (54) and found that the percentage of missing values of a variable, such as clinical status, laboratory results, and clinical actions or procedures, can reach as high as 98%. There is a possibility that this phenomenon might also be observed with data collected through SMART BEAR HAids due to connectivity issue and lack of participant adherence. As a result, simply deleting rows with missing values is not feasible for treating missing data, and imputation and model-based approaches should be used instead. There are several types of both imputation and model-based methods. For imputation methods, there are mean, median, zero, linear interpolation, forward, and backward, whereas for model-based methods, there are linear regression, KNN, and Multiple-value Imputation. A generic method was suggested by Salgado et al. (55) for the purpose of evaluating the performance of various methods for handling missing data. To start with, use a sample of the dataset that contains no missing data as ground truth, and then introduce the proportions of missing data at random in increments of say 5%. In the next step, compute the sum of squared errors (SSE) between the ground truth and the reconstructed data, for each method and for each proportion of missing data. Repeat these steps for each method and calculate the average SSE. Lastly, select the method that performed best at the level of missing data in the given dataset.

In addition, there is the question of how to deal with outliers—“samples that are exceptionally far from the mainstream data” (56). Even with a thorough understanding of the data, outliers can still be difficult to detect (56); however, statistical methods can assist in the identification of them. As standard deviation method is more suited for data with a normal distribution, therefore, it is used after the data have been standardized and normalized. Given the mean and standard deviation of the dataset, z-score can be computed for every ξ_*i*_, which is the number of standard deviations away from the mean, as a way to identify outliers (57). Data points can be declared as outliers if their z-score standard deviation is greater than a predefined threshold. The threshold used in this analysis is three, as it is common practice to identify outliers in data with Gaussian or Gaussian-like distributions.

Lastly, it is important to determine whether there is multicollinearity among the variables. Multicollinearity refers to when there is a lack of orthogonality among two or more variables, and it often creates problems in a regression model (58) because the model results tend to fluctuate significantly when changes are made to independent variables that are highly correlated. In terms of hearing data, multicollinearity is often met among several variables. A typical example is the pure tone thresholds across different frequencies. Pure tone thresholds are measured in frequency bands with each representing a cochlear region, and the neighboring frequencies tend to be highly correlated (59). Moreover, pure tone audiogram also shows a high correlation among the sensitivity of the two ears for each participant when symmetric hearing is present (59). A common method of checking whether the data are multicollinear is to use the Variance Inflation Method (VIF) for each independent variable. In general, a VIF value of 10 indicates weak multicollinearity, and a variable with a higher value is typically considered to have a high correlation with another independent variable (58). A simple way to eliminate high multicollinearity variables is to remove them. However, this may not be feasible in practice. As a result, alternative methods, such as transforming the variables or performing Principal Component Analysis, should be considered instead, depending on the data and the expected outcome. Finally, data will be split into training, validation, and testing sets.

In this conceptual paper, the pre-processing steps discussed here are generic. While these techniques should be considered regardless of the questions to be answered, specific pre-processing methods, such as handling missing data and multicollinearity variables, will only become apparent following the data collection.

#### Hyper-tuning the model

The model is validated on the validation set during hyper-tuning in order to determine the set of optimal hyper-parameters. The hyper-tuning is performed using the Keras Tuner^4^ library to determine the set of optimal hyper-parameters for model trained with TensorFlow^5^. There are many hyper-parameters that need to be determined when training an LSTM model. For this analysis, the number of hidden states in each layer, choice of activation function, learning rate, dropout rate, and batch size are hyper-tuned.

It is imperative to adjust the number of hidden units according to the complexity of the data and select an activation function that is capable of learning the complex relationship in the data. Learning rate is also important because if it is too fast, the model converges too quickly, while if it is too slow, it reaches some local minima. Dropout is a regularization technique while training a DL model, aiming at improving generalization and reducing overfitting. Last but not least, the batch size is the number of samples of training data that will be propagated through the model and should be adjusted accordingly as it impacts the stability of the learning process. Furthermore, the model will also be trained with early stopping in order to prevent overfitting. Early stopping is implemented through a callback function, which monitors the progress of the training, and if no improvements are made during the course of training, the training is terminated early.

#### Proposed model architecture

The proposed prediction model, attn-LSTM, will be trained on the training set with the set of optimal hyper-parameters from hyper-tuning, and the results are reported by predicting the unseen testing set. Table 1 shows the proposed model architecture of attn-LSTM and hyper-parameters setting for each layer. It should note that the choice of learning rate and batch size is hyper-tuned for the entire model and not for each individual layer.

**Table 1:** Proposed model architecture.

**Layer no**.	**Layer description**	**Hyper-parameters setting**
1	Input layer	N/A
2	LSTM layer	Hidden units are hyper-tuned between 32 and 512. Activation function is hyper-tuned between Sigmoid and Tanh.
3	Self-attention layer	N/A
4	Dropout layer	Dropout rate is hyper-tuned between 0.001 and 0.1.
5	Flatten layer	N/A
6	Output (dense) layer	Regression problems: hidden unit is 1, and activation function is hyper-tuned between ReLu, Sigmoid, and None. Binary classification problem: hidden unit is 2, and activation function is Softmax and Sigmoid.

**Figure 4:**
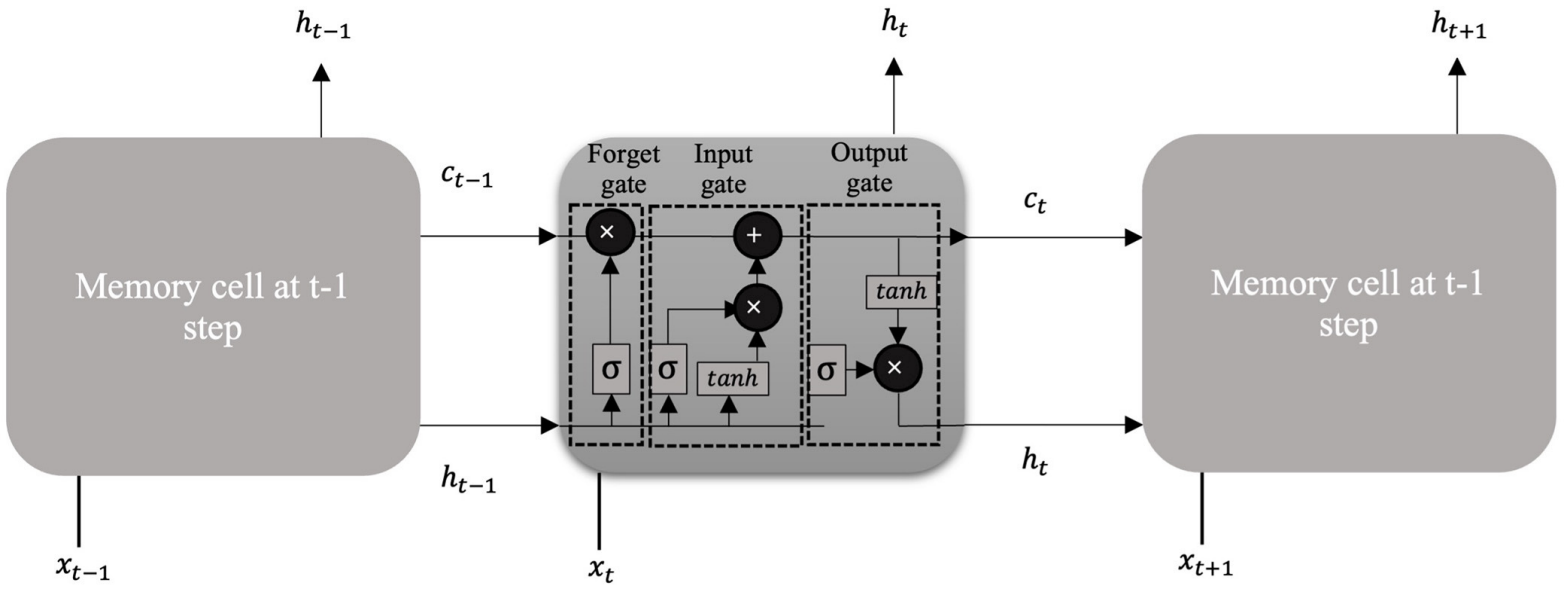
An illustration of the LSTM network.

LSTM (60) is a refined variant of the Recurrent Neural Network that is designed with a feedback architecture such that the current time step prediction is influenced by the network activation from the previous time steps as inputs. LSTM is one of the widely used DL technique for analyzing time series data and is capable of learning long-term time series data as well as short-term time series data (61). The hidden layer inside an LSTM network contains recurrently connected special units called memory cells and their corresponding gate units: input gate, forget gate, and output gate (60) as shown in Figure 4. The input gate is responsible for preventing the memory stored in a memory cell from perturbations by irrelevant inputs. Similarly, the output gate is there so other units are protected from perturbations by currently irrelevant stored memory. To optimize the performance of the LSTM, information that is no longer required by the LSTM is removed in the mechanism of the forget gate.

At each timestep *t*, the cell takes an input vector, *x*_*t*_, and produces an output vector, *h*_*t*_, which also refers to the hidden state of the LSTM. Firstly, the cell needs to determine whether the information from the previous timestep, *t*−1, should be kept or not with the forget gate, *f*_*t*_. The forget gate takes the input vector at current timestep, *x*_*t*_, and the hidden state from the previous timestep, *h*_t*−1_, and produces an output between 0 and 1 where 0 represents “completely forget this information” and 1 represents “completely keep this information”. The forget gate, *f*_*t*_, is calculated as follows:


$$\begin{array}{l} f_{t}=\sigma \left(w_{x}\;x_{t}+w_{h}\;h_{t-1}+b\right)\;, \end{array}$$

where σ is the sigmoid function, *w*_*x*_, *w*_*h*_ are the weighting factor, and *b* is the bias vector. More specifically, the sigmoid function is calculated as:


$$\begin{array}{l} \sigma \left(x\right)=\frac{1}{1+e^{-x}}. \end{array}$$

The next step is to quantify the importance of the new information with the input gate, *i*_*t*_:


$$\begin{array}{l} i_{t}=\sigma \left(w_{x}\;x_{t}+w_{h}\;h_{t-1}+b\right), \end{array}$$

which is also a function of input vector at current timestep, *x*_*t*_, and the hidden state from the previous timestep, *h*_*t*−1_. Then, a new vector named *s*_*t*_ is created which decides if the new information should be stored in the cell state or not. This is done by applying a hyperbolic tangent function, tanh, to the input vector at current timestep, *x*_*t*_, and the hidden state from the previous timestep, *h*_*t*−1_. It is calculated as:


$$\begin{array}{l} s_{t}=\tanh\left(w_{x}\;x_{t}+w_{h}\;h_{t-1}+b\right)\;, \end{array}$$

and the value of new information is transformed to a value between−1 and 1, where−1 means the new information is subtracted from the cell state and 1 means the new information is added to the cell state. The current cell state, *c*_*t*_, is finally updated by taking the previous cell state, *c*_*t*−1_, the forget gate, *f*_*t*_, the input gate, *i*_*t*_, and *s*_*t*_ into consideration by:


$$\begin{array}{l} c_{t}=f_{t}⊙c_{t-1\;}+i_{t}⊙s_{t}\;, \end{array}$$

where ⊙ is the element-wise product. Then, the output gate, *o*_*t*_, determines what information from the cell state is going to be the output. The output gate is also a function of input vector at current timestep, *x*_*t*_, and the hidden state from the previous timestep, *h*_*t*−1_, and outputs a value between 0 and 1. It is calculated as follows:


$$\begin{array}{l} o_{t}=\sigma \left(w_{x}\;x_{t}+w_{h}\;h_{t-1}+b\right)\;. \end{array}$$

Finally, the hidden state, *h*_*t*_, at timestep *t* is updated with the current cell state, *c*_*t*_, and the output gate, *o*_*t*_, by:


$$\begin{array}{l} h_{t}=\tanh\left(c_{t}\right)⊙o_{t}\;. \end{array}$$

The use of attention-based LSTM was initially designed for natural language processing tasks and has been extended to other areas such as computer vision and time series prediction. The attention mechanism is also inspired by the human biological system, such that humans do not process large amounts of data all at once, but instead selectively focus on certain distinct parts of information (62). Moreover, integrating an attention mechanism into an LSTM model architecture may also enhance the interpretability of the model (63), since the attention mechanism can be used to demonstrate which features are important for predicting a particular outcome. The specific attention mechanism adopted in this framework is the Self-attention similar to the one proposed by Vaswani et al. (64), where the mechanism is relating different positions of a single sequence in order to gain a representation of the sequence.

Vaswani et al. (64) introduced a generalized definition for attention functions in which the inputs of the function consist of three vectors: queries (q), keys (k), and values (v). In practice, the attention function is computed on a set of queries simultaneously and packed into the matrix Q, and similarly the keys and values are packed into the matrix K and V, respectively. The concepts of Q, K, and V were first introduced in the context of NLP, specifically with Encoder-Decoder models. Taking the task of machine translation as an example, the query is derived from the Decoder layers reading the current translated text, whereas the key and value are derived from the Encoder layers reading the original sentence.

However, Self-attention is a special case of the attention mechanism where all of the queries, keys, and values come from the same place, such that *Q* = *K* = *V* (64). The mechanism queries only the inputs to obtain the self-attention, and from the self-attention a new representation of the inputs can be constructed. In this framework, the inputs of the attention function are the sequence of hidden state vectors for all timesteps produced by LSTM, *H* = (*h*_1_, *h*_2_, …, *h*_*n*_), therefore, *H* = *Q* = *K* = *V*.

The next step is to calculate a compatibility score for each hidden state vector in the LSTM. More specifically, it involves scoring the compatibility of each hidden state vector in H against the hidden state vector for which the self-attention is calculated. The specific compatibility score used in this framework is similar to the proposed by Vaswani et al. (64) and calculated as follows^6^:


$$\begin{array}{l} \text{Compatibility}\;\text{score}\;=\;\frac{HH^{⊤}}{\sqrt{d_{H}}}\;, \end{array}$$

where *d*_*H*_ is the dimension of the sequence of hidden state vectors and it is a dot-product-based compatibility score. For example, the compatibility score of the first hidden state vector, *h*_1_, is calculated by scoring each hidden state vector, *h*_2_, …, *h*_*n*_, against *h*_1_, with $h_{1}\cdot h_{1}^{⊤}/\;\sqrt{d_{H}},\;h_{1}\cdot h_{2}^{⊤}\;/\;\sqrt{d_{H}},\;$…, $h_{1}\cdot h_{n}^{⊤}\;/\;\sqrt{d_{H}}$. The other commonly used compatibility score is the additive-based one, where the compatibility score is computed using a single hidden layer feed-forward network. Dot-product-based compatibility scores can be space-efficient and much faster in practice when compared to additive-based compatibility scores (64).

Each compatibility score for each hidden state vector is then sent through to the Softmax function in order to normalize the scores so that all scores are positive and sum to 1. Finally, the output of the self-attention function is calculated as a weighted sum of the hidden state vectors and the compatibility score. The matrix of the output is calculated as follows^7^:


$$\begin{array}{l} \text{Attention}\left(H\right)=\text{softmax}\left(\frac{HH^{⊤}}{\sqrt{d_{H}}}\right)H. \end{array}$$

#### Evaluating the model performance

The results of the trained attn-LSTM are reported by predicting the unseen testing set and evaluated using different sets of metrics for classification and regression problems. For classification problems, the evaluation metrics are accuracy, precision, recall, F1 score, and AUC. Accuracy, precision, and recall can be derived from a confusion matrix, and F1 score is the harmonic mean of precision and recall. Each of the metric is calculated as follows:


$$\begin{array}{lll} \text{Accuracy}\; & = & \frac{TP\;+\;TN}{TP+FP\;+\;TN\;+\;FN}\;,\\ \text{Precision}\; & = & \frac{TP}{TP+FP}\;,\\ \text{Recall}\; & = & \frac{TP}{TP+FN}\;,\\ F1\text{ score } & = & 2\;*\frac{\text{Precision }*\mathrm{Recall}}{\text{Precision }+\;\mathrm{Recall}}\;. \end{array}$$

Finally, AUC measures the area under the ROC curve, which is a graphical representation of how well the model performed and shows the relationship between True Positive Rate and False Positive Rate.

For regression problems, four standard error estimators are used, namely Symmetric Mean Absolute Percentage Error (sMAPE), Mean Absolute Scaled Error (MASE), Mean Absolute Percentage Error (MAPE), and Weighted Average Percentage Error (WAPE). The error estimators are calculated as follows:


$$\begin{array}{lll} sMAPE & = & \frac{200}{N}\overset{N}{\underset{t=1}{\sum }}\frac{\left|y_{i}-\tilde{y_{i}}\right|}{\left|y_{i}|+|\tilde{y_{i}}\right|},\\ MASE & = & \frac{1}{N}\overset{N}{\underset{t=1}{\sum }}\frac{\left|y_{i}-\tilde{y_{i}}\right|}{\frac{1}{t+N-1}\overset{t+N}{\underset{j=2}{\sum }}|y_{j}-y_{j-1}|},\\ MAPE & = & \frac{1}{N}\overset{N}{\underset{t=1}{\sum }}\frac{\left|y_{i}-\tilde{y_{i}}\right|}{y_{i}},\\ WAPE & = & \frac{\overset{N}{\underset{i=1}{\sum }}|y_{i}-\tilde{y_{i}}|}{\overset{N}{\underset{i=1}{\sum }}|y_{i}|}, \end{array}$$

where *y*_*i*_ is the true value, $\tilde{y_{i}}$ is the predicted value, and *N* is the number of data points.

Since sMAPE, MASE, and MAPE are percentage-based error estimators, they are scaled-independent so that they can also be used for comparing prediction performance across different datasets. In addition, all error estimators are symmetric, which means that both positive and negative prediction errors are penalized equally. However, MAPE has the disadvantage that the errors tend to blow-up when the variable values are low, causing the results to be misleading. Thus, WAPE is also applied here since the errors are weighted by the total values.

#### Explaining the model

SHAP (41), more specifically, Kernel SHAP, is a local, *post-hoc*, and model-agnostic XAI method that can be used for both classification and regression problems. *Post-hoc* interpretation means that the interpretability is created after the model has been constructed (32) and aims to provide an explanation for the black-box models (65). Another method is ante-hoc, in which the decision-making process or the basis of a technique of a model can be understood by humans without additional information (65). Some of the ante-hoc methods include LR, Decision Tree, and KNN. Both ante-hoc and *post-hoc* methods can be further divided into two approaches, *Model (Global) Explanation* and *Instance (Local) Explanation*. The Local Explanation approach explains only the model prediction for the single data instance, whereas the Global Explanation approach explains the inner workings of the entire model trained on a dataset. Model-agnostic is a subcategory of *post-hoc* methods, such that it can be applied to a variety of models, whereas model-specific can only be applied to one specific type of model.

SHAP uses the Shapley value from Game Theory to assign importance to each feature. In effect, the feature contributions (Shapley values) are calculated by the marginal contribution of the feature over every feature so that how the model behaves in its absence is analyzed, and then the prediction of the model can be written as the sum of bias and single feature contributions (41). According to Lundberg et al. (79), SHAP belongs to the family of *Additive Feature Attribution Methods*, meaning that the Shapley values are applied to binarised features, where a value of 0 corresponds to an unknown feature value, and a value of 1 corresponds to a feature being observed. The explanation model can be written mathematically as:


$$\begin{array}{l} g\left(z^{{\;}^{\prime }}\right)=\phi_{0}+\overset{M}{\underset{i=1}{\sum }}\phi_{i}z_{i}^{{\;}^{\prime }}\;, \end{array}$$

where g is the explanation model of the prediction model, *z*′∈{0, 1}^*M*^ where *z*′is the binarised feature and M is the number of binarised input features, ϕ_0_ is the model output without binarised inputs, and ϕ_*i*_∈*R* are the Shapley values (41). When compared with the other state-of-the-art explanation approach, LIME (36), SHAP satisfies three crucial properties that LIME does not: Local Accuracy, Missingness, and Consistency (41). Local accuracy requires consistency between the outputs of the explanation model and the prediction model. Missingness requires features missing in the original input to have no impact on the output. Lastly, consistency ensures that the impact of a feature does not decrease as it increases or remains the same.

Local accuracy is particularly important for providing explanations, as it ensures that the explanation model is less susceptible to adversarial attacks (66). Adversarial attacks refer to when the outputs of a classifier can be manipulated by a small perturbation of an input to conceal the biases of a system. In the study of Slack et al. (67), the authors attempted to fool both LIME and SHAP in order to determine if the feature contributions can be manipulated through the use of biased classifiers. It was found that the SHAP is less vulnerable to adversarial attacks than LIME due its local accuracy property. It is for these reasons that SHAP was chosen over LIME in our framework.

SHAP is a local XAI method that has been used to explain local predictions in many studies. For instance, Lenatti et al. (42) investigated the contribution of specific feature values to an individual prediction based on SHAP values. It is nevertheless also possible to obtain a global SHAP explanation by calculating the mean absolute SHAP values for each feature across the datasets allowing the global importance of each feature and the relative impact of all features over the entire dataset to be determined.

The results of SHAP will therefore be presented in the form of a visualization, in particular, the summary plots^8^ will be used where it combines the feature importance with feature effects. The x-axis of the plots represents the SHAP value, or the impact on the model prediction, of each feature, the y-axis lists all the features and ordered according to their importance, and the color depicts the value of the feature from low to high.

In addition to the summary plots proposed to be used here, SHAP values can be analyzed in a variety of ways, including a dependence plot to demonstrate the global interaction effects between features. SHAP values may also be useful for assessing the contribution of features to an incorrect prediction, as demonstrated in the work of Lenatti et al. (42).

### Expected outcome and predictors

The objectives of the SMART BEAR project in relations to HL are to answer several questions using the collected SMART BEAR data and the proposed predictive framework that leverages XAI techniques in order to develop a comprehensive profiling of patients with HL. Table 2 summarizes the expected outcome and its associated predictors (characteristics) for each question, and how this framework is applied to each question is discussed below.

**Table 2:** A description of the predictive models, their expected outcome, and associated predictors.

**Prediction models (PM)**	**Predictors**	**Outcome variables**	**Expected outcome**	**Value type**
Q1	Age, biological gender, hearing loss type, hearing loss chronicity, degree of hearing loss, manual adjustments of volume/program, overall HAids satisfaction, time, time of hearing aids usage	Dropout	<45–50%	Y/N
Q2	Age, biological gender, hearing loss type, hearing loss chronicity, degree of hearing loss, time	Time of HAid usage	Adults should use their HAids >10 h a day.	Minutes/day
Q3	Age, biological gender, hearing loss type, hearing loss chronicity, degree of hearing loss, number of visits, manual adjustments of volume/program, time	GHABP score	Described in detail below.	(Integer)
Q4	Age, biological gender, hearing loss type, hearing loss chronicity, degree of hearing loss, overall HAids satisfaction, manual adjustments of volume/program, time, time of hearing aids usage	Number of face-to-face sessions	<4 visits to the Audiologist's in the first 6 months.	(Integer)
Q5	Age, biological gender, hearing loss type, hearing loss chronicity, degree of hearing loss, overall HAids satisfaction, manual adjustments of volume/program, time, time of hearing aids usage	Number of remote sessions	<4 visits to the Audiologist's in the first 6 months.	(Integer)
Q6	Age, biological gender, hearing loss type, hearing loss chronicity, degree of hearing loss, noise exposure, overall HAids satisfaction, time, time of hearing aids usage	Number of manual changes per day	<3 per day.	(Integer)

As mentioned previously, this is a conceptual paper meaning that the precise details of the pre-processing techniques, optimal hyper-parameters for each question, and the prediction and explanation results will only be available once the study is commenced in autumn 2022.

#### Q1—Identification of those characteristics that make patients more prone to drop-out and stop using their HAids

The optimal drop-out rate should be less than the general population with HL (7), therefore, the expected outcome for Q1 is to be <45–50% for aged populations. Clinicians have the option of choosing how the data are aggregated in order to determine what the drop-out rate will be in the future in days, weeks, months, or years. In cases where a weekly analysis is required, for example, the average of HL chronicity, degree of HL, and manual adjustments of volume/program, and the sum of time of HAids usage are calculated for each week to convert the data frequency. Apart from handling missing data, outliers, and multicollinearity among the variables, continuous variables such as age, degree of HL, and time of HAids usage are standardized and normalized, nominal variables such as gender are one-hot encoded, and ordinal variables such as HL chronicity, HL type, and manual adjustment of volume/program are ordinal encoded. In addition, the outcome variable is also treated with label encoding, with 1 representing Yes and 0 representing No, for making a binary classification.

Attn-LSTM is then employed to predict whether or not a participant will stop using their HAids in the future and the identification of characteristics that have an impact on this prediction is carried out through SHAP. Finally, the predicted future number of drop-out participants is compared to the general population with HL in order to compute the drop-out rate.

#### Q2—Identification of those characteristics that make patients more prone to use their HAids sufficiently long during the day

It is recommended that adults should use their HAids for more than 10 hours a day (76). Due to this, data are aggregated to have a daily frequency by default. This is done by taking the average of HL chronicity, degree of HL, manual adjustments of volume/program, and overall HAids satisfaction for each day, and the sum of time of HAids usage for each day in minutes. It should note that, although the data are transformed to have a daily frequency by default, clinicians will still have the option to choose to analyse monthly HAid usage, for example, if required. Similarly to Q1, continuous variables are standardized and normalized, while nominal and ordinal variables are one-hot and ordinal encoded, respectively. Missing data, outliers, and multicollinearity will also be treated with appropriate pre-processing techniques.

As a regression problem, attn-LSTM is used to predict participants' future HAids usage. SHAP is then used to interpret the model prediction to identify which characteristics influence participants to use their HAids more often.

#### Q3—Identification of those factors augmenting the benefit of patients from using their HAid

The Glasgow Hearing-Aid Benefit Profile (GHABP)^9^ is a questionnaire that was designed to assess the operational management for HAid benefit, both at the systematic and clinical levels (15). The questionnaire will assess 4 situations with 6 questions, which are scored with 1 being the best score and 5 being the worst score. Whitmer et al. (77) recruited 1,574 participants and were asked to rate their hearing disability, handicap, HAid use, HAid benefit, HAid satisfaction, and residual (aided) disability with the GHABP questionnaire. The participants were divided into none, unilateral, and bilateral aided users and assessed in the four situations: quiet conversations, TV listening, noisy conversations, and group conversations. Their findings regarding the normative GHABP score for HAid benefit will be used as the expected outcome for Q3.

Q3 is also a regression problem as the future GHABP score is predicted with attn-LSTM, and the reasons for this prediction are provided by SHAP. When clinicians require a monthly analysis, for example, the average of the GHABP score, HL chronicity, degree of HL, number of visits, and manual adjustments of volume/program, and the sum of time of HAids usage are calculated for each month to convert the data frequency. For Q3, pre-processing steps are similar to those used for previous questions, where continuous variables such as age, degree of HL, and time of HAids usage are standardized and normalized, nominal variable such as gender are one-hot encoded, and ordinal variables such as GHABP score, HL chronicity, HL type, number of visits, and manual adjustments of volume/program are ordinal encoded.

#### Q4—Identification of those factors decreasing the number of needed face-to-face sessions with their audiologist for counseling and/or HAid fine tuning, as an indicator of better self-management and optimal initial HAid configuration

The number of face-to-face with the audiologists is suggested to be <4 times in the first 6 months (78). Following this, the data are transformed to have a monthly frequency by default, with the options of analyzing the data at other frequencies still available. Therefore, the average of HL chronicity, degree of HL, number of visits, overall HAids satisfaction, and manual adjustments of volume/program, and the sum of time of HAids usage are calculated for each month. Nominal variables such as gender are one-hot encoded, ordinal variables such as overall HAids satisfaction, HL chronicity, HL type, number of visits, and manual adjustments of volume/program are ordinal encoded, and continuous variables such as age, degree of HL, and time of HAids usage are standardized and normalized.

As a regression problem, the future number of face-to-face sessions is predicted using attn-LSTM, and the characteristics affecting the prediction are investigated with SHAP.

#### Q5—Identification of those factors decreasing the number of needed remote sessions with their audiologist for counseling and/or HAid fine tuning, as an indicator of better self-management and optimal initial HAid configuration

Similar with Q4, the suggested number of remote sessions with the audiologists is also to be <4 times in the first 6 months (Tecca, 2018). Therefore, the default frequency is also set to be monthly, and attn-LSTM is used to predict the number of remote sessions with the audiologists in future months. SHAP is then used to identify the characteristics that influence participants to request fewer sessions with their audiologist. The pre-processing steps are also in line with Q4.

#### Q6—Identification of those factors decreasing the number of manual changes of HAid program, as indication of poor sound quality and bad adaptation of hearing aid configuration to patients' real needs and daily challenges

Although there is no precise definition for the optimal number of manual adjustments of the HAids, clinical experience has shown that fewer than three manual changes per day is considered as acceptable. By default, data are transformed to have a daily frequency in order to predict future daily manual adjustments with attn-LSTM, with SHAP providing information on the characteristics that impact the prediction.

It is also possible for clinicians to select a different data frequency for this analysis if required. The average of HL chronicity, degree of HL, number of visits, overall HAids satisfaction, and manual adjustments of volume and program, and the sum of time of HAids usage are calculated for each day to convert the data frequency. Pre-processing steps also consists of handling missing data, outliers, multicollinearity. As well as transforming continuous variables with standardization and normalization, ordinal variables with ordinal encoding, and nominal variables with one-hot encoding.

As a final point, SHAP values are analyzed with the same principle for all questions. The y-axis on the SHAP summary plot would indicate the most important feature on average for attn-LSTM to predict a certain outcome. The x-axis, along with the color, would show the impact of each feature value on the model prediction. For example, the SHAP values for Q1 may indicate that perhaps Age is the most important feature on average for participants to stop using their HAids. More specifically, younger participants might be less likely to drop out, whereas perhaps participants with a lower HAids usage might be more likely to stop using their HAids. As for Q3, SHAP result might show that perhaps HL type influences future GHABP score the most on average, where participants with a mixed type of HL might be more likely to benefit from their HAids.

## Results—Discussion

This paper is a conceptual paper that synthesizes previous work on prediction models in healthcare and audiology (20, 27, 30, 31), and further describes the design and methods of the Big Data research project SMART BEAR with which we are aiming to fill the identified knowledge gaps. To the best of our knowledge, SMART BEAR represents the first research initiative in hearing research aimed at integrating such large and heterogeneous datasets and analyzing them using AI and XAI methods.

According to Mellor et al. (12), many factors beyond the pure tone audiogram should be monitored and dynamically adapted in order to achieve optimal hearing rehabilitation. Prognostic prediction models using audiometric and other lifestyle or medical data may be helpful toward achieving this goal. Education level (68), cognitive performance (69), and performance on speech recognition tests (70) have previously been suggested as potential prognostic factors. Following this, a wide range of data is collected in SMART BEAR as shown in Supplementary materials 2, 3, such as demographics, audiometric data, cognitive status, mental status, habits, and biological gender. Taking advantage of the ability of modern HAids to record their dynamic operation will also enable a relatively low-cost collection of data, such as hours of HAid use, from a large population, while clinical assessment will provide insight into the clinical context of the collected data. Furthermore, instead of assessing patients in a laboratory environment, SMART BEAR is collecting data both at the office and in real life through clinical assessments and smart sensors.

The created and continuously updated data can then be viewed as sequences with temporal elements and contain high-dimensional clinical variables (63). Therefore, collected SMART BEAR data will be analyzed through time-dependent multivariate prediction models that are capable of handling both classification and regression problems while ensuring a high level of accuracy. The XAI method will then be applied in order to explain the model to clinicians so that they will be able to better understand how the model arrives at the predicted results. In this study, attention-based LSTM is proposed to be the prediction model and then using SHAP to interpret the model. The proposed framework introduced in this conceptual paper can also be applied to other comorbidities within the SMART BEAR project.

The findings of this analysis will have implications in clinical practice, health policies and research.

### Clinical and research implications

With proper analysis and interpretation of SMART BEAR results, the most accurate patient profile to date can be created for HL patients, allowing it to serve as a valid proxy for anticipated behavior even before the initial HAid fitting session. According to the analysis of synthetic hearing data conducted within the context of the H2020 project EVOTION^10^, higher levels of physical activity are associated with longer daily HAid use (43). Therefore, SMART BEAR results also aim to provide a better understanding how physical activity, such as walking, affects HAid experience in order to incorporate physical activity promotion into hearing rehabilitation for different populations. Furthermore, different factors relating to hearing rehabilitation might be identified with different participants. This is shown in the data-driven analysis with the subjective data of 572 HAid users conducted by Sanchez-Lopez et al. (71), where participants with different HL degree preferred different types of hearing rehabilitation. Other factors may include presence of particular comorbidities or different living situations, therefore, the combinations and interactions between the factors will also be examined in SMART BEAR.

The patient profiling proposed by SMART BEAR may be able to assist manufacturers and clinicians in making optimal choices in terms of HAid model and configuration options, or, in future stages, it could create automatic fine-tuning of HAids (12). In this context, after the end of the study, SMART BEAR is considering providing access, upon request, to the de-identified dataset for future exploration. Participants will be fully informed and will provide their consent so access to their de-identified data can be granted in the future for specific scientific purposes. Open Access will be provided for the following SMART BEAR datasets: anonymised data from demographics, questionnaires, interviews, anonymised sensor raw data, video of the protocols for annotation, and anonymised data from basic clinical information for annotation. It is envisaged that this policy will facilitate the use of SMART BEAR's gained knowledge by a range of different stakeholders.

### Limitations

All participants in SMART BEAR will be fitted with the same HAid model, following the same fitting protocol, with the use of the same algorithm. Although the fine-tuning and the program selection of the HAids will be based on the needs and preferences of each participant, the fitting of the HAids may not be optimal for every participant when only one universal fitting protocol is used. However, this choice was made since the comparison of programs or algorithms is not in the scope of SMART BEAR, as well as in order to avoid unnecessary heterogeneity or lower quality of the data as a result of systematic errors. This limitation will be taken into account in the interpretation of our results. Moreover, SMART BEAR participants will only be between the ages of 67 and 80, which means that its results cannot be generalized to a population younger than that. Data like hours of usage and changes in programs will be subject to connectivity loss, which is a significant barrier in similar projects (50). The impact of loss of follow-up patients, such as the unavailability of information regarding continuation of usage, is also expected to be low, provided that this percentage will remain in the predicted range (below 20%). Close follow-ups and dedicated helpdesks will help minimize these risks, while imputation and model-based approaches will facilitate dealing with missing data, as explained above. Another limitation will be the variation in the population between six different countries with socioeconomic and cultural diversities; however, comparison between study groups is expected to produce useful results. Finally, speech audiometry in quiet or in noise is not part of the SMART BEAR data collection. This is due to the fact that there do not currently exist any universally validated materials that could be used across all six countries and thus in all languages. Speech audiometry, while recognized as having clinical value in fitting choices, does not fall under the scope of SMART BEAR. As an alternative approach to assess HAid benefit, we are aiming to collect other parameters, including real-life data, such as hours of usage and manual changes of programs, as well as interview data, such as the GHABP questionnaire.

It is noteworthy that unlike the evaluation metrics used in this paper to evaluate a prediction model, there are currently no widely accepted objective metrics for evaluating XAI methods. Though the proposed XAI method will be validated by clinicians and medical experts in SMART BEAR, this will only provide a subjective assessment of the XAI method. To this end, existing evaluation metrics for XAI metrics, such as Rosenfield's set (72), should be tested in the future with the collected data in order to obtain both objective and subjective validation. Although SHAP is one of the best known XAI methods, it is often criticized for long computation time and Shapley values do not work if features are correlated (73). As a result, the proposed framework may be unable to deliver what clinicians require in cases where the characteristics to be identified are correlated. Therefore, alternative methods of XAI should be considered in the future. Among them is Attention Mechanism-based XAI methods, such as the one proposed by Choi et al. (74) and Schockaert et al. (75). An attention mechanism-based XAI method can provide an explanation for Recurrent Neural Network or its variants by assigning corresponding values to the importance of the different sub-sequence of the input sequence according to the model and may be more suitable for the proposed prediction model.

## Conclusion

SMART BEAR is, to the best of our knowledge, the first big data study whose goal is to integrate heterogeneous and contextualized HAid, medical, societal, and environmental data in order to develop and validate a prognosis framework using AI and XAI methods. The outcomes of the project are expected to benefit multiple stakeholders in the field of Audiology, such as HAid users, manufacturers, clinicians, researchers, and health policy makers, as well as to influence current practice and future research. These outcomes could also improve confidence in integrating AI models in the medical field, particularly with encouraging AI to be used in the medical decision-making process by utilizing XAI methods to enhance its interpretability, transparency, and accountability.

## Ethics statement

This is a conceptual paper describing the rationale and design of the large scale H2020 project SMART BEAR. The SMART BEAR protocols have obtained, or are in the process to obtain, ethical approval in all six countries. All participants will have to provide their voluntary consent after oral and written information about the details of the project. General Data Protection Regulation (EU) 2016/679 (GDPR) principles will be implemented in all stages of data collection, storage and sharing.

## Author contributions

EI, QS, CK, and TB: conceptualization, methodology: writing—original draft, and writing—review and editing. DK: writing—original draft. CK and TB: supervision. All authors contributed to the article and approved the submitted version.

## Funding

SMART BEAR was funded by the European Commission (Grant Agreement No.: 857172/H2020-SC1-FA-DTS-2018-2).

## Conflict of interest

The authors declare that the research was conducted in the absence of any commercial or financial relationships that could be construed as a potential conflict of interest.

## Publisher's note

All claims expressed in this article are solely those of the authors and do not necessarily represent those of their affiliated organizations, or those of the publisher, the editors and the reviewers. Any product that may be evaluated in this article, or claim that may be made by its manufacturer, is not guaranteed or endorsed by the publisher.

## Footnotes

^1^ https://www.who.int/news-room/fact-sheets/detail/deafness-and-hearing-loss

^2^ https://www.smart-bear.eu/

^3^ https://www.thebsa.org.uk/wp-content/uploads/2018/11/OD104-32-Recommended-Procedure-Pure-Tone-Audiometry-August-2018-FINAL.pdf

^4^ https://keras.io/keras_tuner/

^5^ https://www.tensorflow.org/

^6^The original notation for the generalized compatibility score in Vaswani et al. (64) is $\frac{QK^{⊤}}{\sqrt{d_{k}}}$.

^7^The original notation for the generalized output of the attention function in Vaswani et al. (64) is $Attention\left(Q,\;K,\;V\right)=softmax\left(\frac{QK^{⊤}}{\sqrt{d_{k}}}\;\right)V.$

^8^ https://shap-lrjball.readthedocs.io/en/latest/generated/shap.summary_plot.html

^9^ https://www.hey.nhs.uk/wp/wp-content/uploads/2020/09/HEY1167-2020-GHABP.pdf

^10^ https://h2020evotion.eu/
