# Supplementary material for: Profiling hearing aid users through big data explainable artificial intelligence techniques
Source: Front Neurol. 2022 Aug 26;13:933940. doi: 10.3389/fneur.2022.933940 (PMC9459083; doi:10.3389/fneur.2022.933940)
Supplement: Supplementary file 1 [file Table_1.DOCX]

Supplementary Material

# Supplementary Table 1. Variables and covariates that will be collected through SMART BEAR HAids.

| **HL related Covariates** | **Acronym** | **Description** | **Value type** | **Source of data** |
| --- | --- | --- | --- | --- |
| Time of measurement | TIME | timestamp at 6 and 12 months | Timepoint | HAid |
| Time | TIMEC | Time as continuous variable in order to link each data item to specific time point | Continuous | HAid |
| Age | AGE | Years of age | Ratio | Clinician’s dashboard |
| Biological gender | SEX | Female or Male | Dichotic | Clinician’s dashboard |
| Hearing loss type | HLTYPE | Predefined text for specific types of hearing loss (Sensorineural, conductive, mixed) | Categorical | Clinician’s dashboard |
| Hearing loss chronicity | HLCHRNCTY | Years since diagnosis of hearing loss | Years | Clinician’s dashboard |
| Side of hearing loss | SIDE | Right, left, bilateral | Categorical | Clinician’s dashboard |
| Ear side | EAR | Right/left (in order to correspond to PTA thresholds) | Categorical | Clinician’s dashboard |
| Fitting side | FIT | Right/left/bilateral | Categorical | Clinician’s dashboard |
| Degree of hearing loss | HLDGREE | Predefined text for clinician to choose according to participant’s pure tone audiogram | Categorical (mild, moderate, moderate-severe, severe)* | Clinician’s dashboard |
| Baseline pure tone average threshold for right ear* | PTAR | Mean value of PTA threshold at 0.5-4kHz (right ear) | Continuous | Clinician’s dashboard |
| Baseline pure tone average threshold for left ear* | PTAL | Mean value of PTA threshold at 0.5-4kHz (left ear) | Continuous | Clinician’s dashboard |
| Baseline pure tone threshold per frequency for right ear* | PTAR_0.5-8kHz_ | Value of PTA threshold at 0.5-8kHz (right ear) | Continuous | Clinician’s dashboard |
| Baseline pure tone threshold per frequency for left ear* | PTAL_0.5-8kHz_ | Value of PTA threshold at 0.5-8kHz (left ear) | Continuous | Clinician’s dashboard |
| Drop out | DROP | Dropout of HA usage | Dichotic | Clinician’s dashboard |
| Time of HA usage | HAUSE | Average time of usage per day till that particular point | Continuous | HAid |
| Number of visits to audiologist’s office | VISITS | Number of necessary visits to the audiologist’s office | Integer | Clinician’s dashboard |
| Overall HA usage satisfaction | GHABP | Total score on GHABP and per situation | Integer | HAid |
| Percentage of usage per environment | ENVRNMT% | Average percentage of time spent in the predefined environments per day | % | HAid |
| HA hourly usage per environment | ENVRNMT | Hourly hearing aid usage (in minutes per hour) from the momentary sound environment encountered by the users. | Integer | HAid |
| Noise Exposure | NOISE | Average noise exposure per day (dB SPL x Time  AND  dB TWA) | Continuous | HAid |
| Manual adjustments of volume | VOLUME | Average number of manual adjustments of volume by the participant | Integer | HAid |
| Manual adjustments of program | PROGRAM | Average number of manual adjustments of programs (already loaded on the HA) by the participant | Integer | HAid |

* Humes LE. The World Health Organization's hearing-impairment grading system: an evaluation for unaided communication in age-related hearing loss. Int J Audiol. 2019 Jan;58(1):12-20. doi: 10.1080/14992027.2018.1518598. Epub 2018 Oct 15. PMID: 30318941; PMCID: PMC6351193.
