# Supplementary material for: Profiling hearing aid users through big data explainable artificial intelligence techniques
Source: Front Neurol. 2022 Aug 26;13:933940. doi: 10.3389/fneur.2022.933940 (PMC9459083; doi:10.3389/fneur.2022.933940)
Supplement: Supplementary file 2 [file Table_2.DOCX]

Supplementary Material

# Supplementary Table 2. Medical assessment conducted for all comorbidities during the baseline visit and repeated at 6 and 12 months after the entry to the study. Data will be collected through the clinician’s dashboard. Specific questionnaires such as the PHQ-9 or Single Item Sleep Quality Scale Score will be administered via the SMART BEAR App every two weeks.

| Assessment | Procedure | Description | Data Points | Data Collection Frequency |
| --- | --- | --- | --- | --- |
| General Assessment  45’ | Demographics | Age, Birth Gender, Source of referral, Ethnicity, Education level, Living situation | 6 | 1-3 (*) |
|  | Medical history + medication use | Diabetes (Prediabetes, Diabetes type I, Diabetes type II on diet, Diabetes type II on insulin),  Hearing Loss (aided/unaided),  CVD history,  Cognitive issues,  Weight Loss,  History of substance abuse,  History of brain injury/stroke,  Depression Disorder,  Anxiety disorder,  Other medical history,  Current medication use (name, dose),  Mini nutritional assessment MNA questionnaire | 1  1  1  1  1  1  1  1  1  >1  >1  20 | 1-3 |
|  | Physical examination | Body height,  Waist circumference,  Hip circumference,  Body weight,  salt intake (high, average, low),  Abdominal palpation (ascites, hepato/splenomegaly),  Peripheral arteries palpable or nonpalpable | 1  1  1  1  1  >1  1 | 3 (**) |
|  | Diet supplement use | Name, dose | >1 | 1-3 |
|  | Godin leisure-time exercise questionnaire | Activity indicator (assessment of number of specific exercises are performed on average for more than 15 minutes during a typical week, self-reported): Scores | 4 | 3 |
|  | RGA - Rapid Geriatric Assessment | Rapid assessment of medical status, functional capabilities, cognitive status, and psychosocial structure and support, self-reported: Scores | 7 | 3 |
|  | IADL | Assessment of ability to perform everyday tasks i.e., food preparation, self-reported. Scores | 27 | 3 |
|  | Geriatric Depression Scale (GDS-short form) | 15-item assessment of level of enjoyment, interest, social interactions, self-reported: Scores | 16 | 3 |
|  | Euro Quality of Life (EQ-5D) | Standardised measure of health-related quality of life, self-reported: Scores | 5 | 3 |
|  | MOCA test | Screening assessment (interview) for cognitive decline: Scores and sub scores | 14 | 3 |
| Frailty  10’ | Edmonton Frailty Scale (EFS) | Assessment of frailty levels, self-reported: Scores | 10 | 3 |
| Cardiovascular Disease (CVD)  20’ | Number of Visits to the ER due to hypertension peak | Self-reported number | 1 | >3 (***) |
|  | Heart osculation | Obtained during the clinical assessment by the SMART BEAR Clinician: Normal, abnormal, murmur | >1 | 3 |
|  | Single item Sleep Quality Scale Score | 1-item assessment of sleep quality, self-reported: Score | 1 | Every two weeks |
|  | Tobacco | Units per day | 1 | 3 |
|  | Systolic Blood Pressure | Standing Blood pressure systolic,  Supine Blood pressure systolic,  Arm from which pressure was taken (right, left) | 2 | 3 |
|  | Diastolic Blood Pressure | Standing Blood pressure diastolic, Supine Blood pressure diastolic,  Arm from which pressure was taken (right, left) | 2 | 3 |
|  | Heart Rate | Heart Rate after subject has been lying down for 3-5 minutes | 1 | 3 |
|  | ECG | Diagnosis | 1 | 3 |
|  | Hb A1c level | Retrospective information, if available | 1 | 3 |
|  | LDL/HDL cholesterol level | Retrospective information, if available | 1 | 3 |
|  | Score CV risk | Percentage | 1 | 3 |
| Mental Disorders  15’ | PHQ-9 | Scores | 10 | Every two weeks |
|  | Single-item Sleep Quality Scale (SQS) | Scores | 1 | Every two weeks |
| Hearing Loss  2h (and 30’ every follow up) | GHABP | Assessment of HAid benefit in specific everyday life situations (interview): Score of part 1 and, after HAid fitting, part 2 | >3 | >3 |
|  | Noise exposure history | Units, structured interview | 4 | >3 |
|  | Otoscopy | Normal/abnormal | 1 | >3 |
|  | Tympanometry | A, B, C and Volume, Peak pressure, Compliance | 4 | >3 |
|  | Pure Tone Audiometry | Air and Bone threshold per frequency and per ear | 18 | 1-3 |
| Balance Disorders  30’ | MiniBEST- test | 14-item clinical assessment of dynamic balance components including anticipatory postural adjustments, reactive postural control, sensory orientation and dynamic gait: Scores | 15 | >3 |
|  | Functional Gait Assessment | 10-item clinical assessment of performance of complex gait tasks: Scores | 10 | >3 |
|  | Rapid Assessment of Physical Activity | 9-item for assessment of physical activity, self-reported: Scores | 9 | >3 |
|  | Falls Efficacy Scale International (FES-I) | Assessment of level of concern regarding falling during social and physical activities, self-reported: Scores | 8 | >3 |
|  | The Activities-specific Balance Confidence Scale | Assessment of patient’s perceived confidence for performing 16-activities of daily living without losing balance, self-reported: Scores | 17 | >3 |

**(*) 1 – 3:** refers to that these variables will only be collected once during the baseline visit (time point 1) if no changes are reported. However, since these variables are monitored via the clinician’s dashboard during the 12-month period, data will be collected again at 6 and 12 months (time points 2 and 3) after the entry into the study if any changes are reported.

**(**) 3:** refers to the total number of time points that these variables will be collected, respectively at time points 1, 2, and 3.

**(***) > 3:** refers to that the minimum number of time points for collecting these variables will be at least three, respectively at time points 1, 2, and 3. During the 12-month period, these variables are also monitored via the clinician’s dashboard. If any changes are reported, then data will be collected again.
