# Supplementary material for: Profiling hearing aid users through big data explainable artificial intelligence techniques
Source: Front Neurol. 2022 Aug 26;13:933940. doi: 10.3389/fneur.2022.933940 (PMC9459083; doi:10.3389/fneur.2022.933940)
Supplement: Supplementary file 3 [file Table_3.DOCX]

Supplementary Material

# Supplementary Table 3. Variables and covariates that will be collected through SMART BEAR sensors.

| **Variable** | **Acronym** | **Description and metric** | **Type** | **Data Collection Frequency** | **Source** |
| --- | --- | --- | --- | --- | --- |
| Diastolic Blood Pressure | DIASTLC | mmHg | [Integer] | Twice per day | Blood pressure tracker |
| Systolic Blood Pressure | SYSTLC | mmHg | [Integer] | Twice per day | Blood pressure tracker |
| Heart Pulse | PULSE | Number of heart beats per minutes at rest | [Integer] | Twice per day | Blood pressure tracker |
| Irregular Heartbeat | IRR | Detection of irregularity in heart rate | [Integer] 0 = not present, 1 = present | Whenever applicable | Smart watch |
| Number of Episodes of Irregular Heartbeat | IRRN | Number of episodes of irregularity of heartbeat till that time point | [Integer] | Once per day | Smart watch |
| Body temperature | Tbody | Body temperature | °C, integer | Twice per day | Thermometer |
| Physical activity (steps per day) | STEPS | Number of steps in 24h | integer | Once per day | Smart watch |
| Physical activity | ACTVT | Minutes of physical activity per day | integer | Once per day | Smart watch |
| Body weight | BW | Timestamped AND Average body weight per week | kg, floating point | Once per day/week | Scale |
| Body fat | BF | Timestamped AND Average Body fat per week | %, floating point | Once per day/week | Scale |
| Body Mass Index (BMI) (advertised) | BMI | weight / (height * height), Timestamped AND average per week | Floating point | Once per day/week | Scale |
| Body water | BWATER | Timestamped AND average per week | %, floating point | Once per day/week | Scale |
| Body lean mass (advertised) | BLM | Timestamped AND average per week | kg, floating point | Once per day/week | Scale |
| Body muscle mass | BMM | Timestamped AND average per week | kg, floating point | Once per day/week | Scale |
| Blood oxygen saturation | Bloodoxygen | Timestamped and average per day and per week | %, integer | Twice per day | Oximeter |
| Number of desaturation episodes | DESATURATION | SaO2<92% episodes | integer | Once per day/week | Oximeter |
| Active kilocalories (dietary calories) burned through actual movement and activity during the monitoring period. | "activeKilocalories" | Average per day | kCal, integer | Once per day/week | Smart watch |
| Cumulative duration of activities of moderate intensity, lasting at least 600 seconds at a time. Moderate intensity is defined as activity with MET value range 3-6. | "moderateIntensityDurationInSeconds" | Average per day | seconds, integer | Once per day/week | Smart watch |
| Minimum of heart rate values captured during the monitoring period, in beats per minute. | "minHeartRateInBeatsPerMinute" | Per day | BPM, integer | Once per day/week | Smart watch |
| Average of heart rate values captured during the last 7 days, in beats per minute. The average heart rate value for the monitoring period can be calculated based on the data from | averageHeartRateInBeatsPerMinute" | Per week | BPM, integer | Once per day/week | Smart watch |
| Average heart rate at rest during the monitoring period, in beats per minute. | "restingHeartRateInBeatsPerMinute" | Timestamped | BPM, integer | Once per day/week | Smart watch |
| Hue light level (Hue motion sensor) | hueLightLevel | Timestamped | % | Once per day/week | Home sensors |
| Hue light illuminance (Hue motion sensor) | hueLightIllu | Timestamped | % | Once per day/week | Home sensors |
| Room temperature (Hue motion sensor) | roomTemp | Timestamped | % | Once per day/week | Home sensors |
